# Black Snow

**Published:** 1868-08

**Authors:** 


BLACK SNOW.—Dr. Andrews, in the *Detroit Review of Medicine and Pharmacy*, describes a phenomenon which occurred on the twenty-fourth of February, in the neighborhood of Romeo, Mich. At the conclusion of a snow storm there fell half an inch of dirty looking snow, which was proved to owe its color to particles of silicious matter, mingled with fibres and mutilated cells, apparently the *debris* of vegetation. He estimated the amount of the extraneous matter to be a grain to the square foot, which would make nearly two tons to the square mile.

